# History of a Case of Human Rumination; with an Inquiry into the Nature of the Process, and into Some of the Phenomena of Digestion. With an Historical Relation of Similar Affections

**Published:** 1821-05

**Authors:** James Copland

**Affiliations:** Member of the Royal College of Physicians of London.


*History of a Case of Human Rumination; with an Inquiry into the Nature of the Process, and into some of the Phenomena of Digestion. With an Historical Relation of similar Affections.* By James Copland, M.D. Member of the Royal College of Physicians of London. (In a Letter to Dr. Hutchinson.)

PERCEIVING, in your Proëmium for the second semi-annual period of the last year, a case of human rumination referred to, and having lately had a similar affection brought to my notice, in an old and intimate friend, I am induced, from the great attention you have paid to physiological subjects in the direction of your Journal, to submit the particulars of this case, with some observations on the nature of the affection, to your disposal.

The novelty of such cases, and their physiological relations, led me to pay as much attention as was in my power to the particular phenomena constituting this affection; and the kindness and intelligence of my friend afforded me great facility in obtaining every information that I desired.

The subject of this case is a gentleman in the meridian of his age, of a strong but spare habit, and of the sanguineo-melancholic temperament. Owing to causes to which he was subjected through the very early period of life, he had been obliged to take his meals in a very hasty manner. This was continued through the subsequent periods of early age, partly from the circumstances to which he was subjected, and in part from the activity of his mind in accomplishing whatever he was engaged in, whether serious employments or the pursuit of pleasure. The very few minutes allowed to his ordinary meals led to a hasty and imperfect mastication of the food; and, although his time was at his own disposal as he reached manhood, still the habit has been retained through the rest of the already passed portion of his life.

The greater part of its early period was spent in an active, varied, and pleasant employment, generally in the open air, and in the vicinity of the sea; and every moment that could be procured from his necessary avocations, was directed to study. This alternation of active exercise, in so healthy a situation, with mental exertion and periods of sedentary application, preserved the due equilibrium of the organic actions; the former neutralizing the effects produced upon the digestive functions by the co-operation of an hasty and imperfect mastication of the ingesta, and by sedulous study.

So long as this diversified mode of living was enjoyed, the regular operation of the digestive tube was continued, and no symptoms of dyspepsia appeared, until he took up his constant residence in the metropolis.

The dyspeptic symptoms appeared several years since, and at the commencement were only occasional. Flatulence, acidity, and cardialgia, chiefly prevailed; but at no time did he experience any loss of appetite, which through life has been uniformly good; nor at any period has he had to complain of any serious malady.

For a considerable time, the chief and almost only complaint was apepsia, or water-qualm: for two or three hours after every considerable meal, part of the more liquid contents was ejected from the stomach, in large mouthfuls, at intervals of from two to five or ten minutes, attended with a slight acidity; sometimes with a slight flatulence and sense of fullness at the stomach, but never with any cardialgia, nor with the slightest sensation of nausea. This affection was generally augmented by any of the usual articles of dessert, or by port-wine; while it was relieved, or entirely prevented, by a moderate quantity of white wine during and after dinner, and by avoiding every species of exertion that could tend to disturb the function of digestion. This affection, after continuing several years, with occasional interruptions, according to the care and means taken to prevent it, passed at last into complete rumination, which has been present after every considerable meal for some time. But, as it was attended with less inconvenience than the preceding apepsia, and being unaccompanied with any disagreeable sensation, no great importance was attached to it, until it became complicated with a cutaneous eruption.

For that I was consulted; and, upon making inquiry into the state of the digestive organs, was readily informed of the ruminating affection. The professional intercourse that now took place furnished me with the particulars already related, which may serve as an introduction to a knowledge of the nature of this disease.

The following is a statement of the particulars of this case, when submitted to my care; for which I am considerably indebted to my intelligent friend.

The cutaneous eruption just alluded to, and to which my attention was first directed, owing to its being the chief object of concern with my patient, was the *lepra vulgare* of Willan; affecting the skin, to the extent of an hand-breadth, on the inner flexure of the knee-joint. It had been of two or three months' duration, and, with the exception of its apparent commencement in the same situation of the elbow-joint, it was present on no other part of the body. The ruminating affection was at that time generally present after all his meals, and constantly after breakfast and dinner.

The appetite was always good, and the food constantly taken in large mouthfuls, was masticated hastily and imperfectly, and swallowed eagerly. There was no thirst. Bowels were habitually costive. His sleep was sound.

His meals were taken more with a desire to satisfy an unpleasant sensation or a requisite desire, than to indulge the pleasures of the palate, and was performed hastily, in order that the studies and pursuits, to which he considered eating an interruption, might be immediately resumed.

Under the usual circumstances, rumination commenced from a quarter of an hour to an hour and a half after a meal. Immediately upon the commencement of this act, a slight sensation of fulness might be felt at the cardia, when the attention was particularly directed to it, that led to a deeper inspiration than usual. So soon as the act of inspiration was completed, and while the muscles of the glottis remained fixed, a bolus of the unchanged aliment rose rapidly from the stomach, with the first effort at respiration, at the moment when the diaphragm had just relaxed, and the re-action of the abdominal muscles commenced. But expiration did not take place until the alimentary ball had passed completely into the mouth, as the glottis remained closed until then: upon this having taken place, expiration was immediately effected; and so rapidly did expiration succeed to the regurgitation of the alimentary bolus, that the latter (unless when the attention was closely applied to the subject,) appeared as part of the expiratory act.

The ruminating process was never accompanied, at any time, with the smallest degree of nausea, nor any pain or disagreeable sensation. The returned alimentary bolus was attended with no unpleasant flavour, was in no degree acidulous, and was equally agreeable, and was masticated with additional pleasure, and with much greater deliberation than when first taken.

The whole of the aliments received at any one meal was not returned in order to undergo this process, only the part that had undergone an insufficient mastication; which indeed constituted the greater portion of solid aliment. That taken at the commencement of a meal was the first disgorged: this was ascertained by eating from a variety of solid dishes, or from partaking of different portions of the same. The more fluid portions were not always returned, unless along with the more solid or imperfectly masticated parts. When, however, the stomach was distended by a large meal, the fluid contents were frequently returned, and subjected to this process.

This affection may be considered as having been passively under the control of the will; and, although it sometimes took place when nearly unconscious of the process, yet it never occurred when the mind was incapable of being acted on by external impressions received by the senses. Thus, if at any time, from previous fatigue, and the concentration of the organic nervous energy towards the digestive organs, sleep was induced immediately after a full meal, this affection did not take place; but flatulence, acrid eructations, &c. afterwards supervened, and continued for some time, in consequence of the gastric juices being insufficient to the production of the requisite changes on the ingesta retained in a state of imperfect division. Very frequently, when the ruminating process was thus prevented, or voluntarily suppressed when circumstances required it, the ingesta, both fluid and solid, were returned at the end of several hours; but were then generally acid, frequently acrid and bitter, and sometimes in so large a quantity as to fill the mouth beyond its capacity of retention. But even then no cardialgia nor gastrodynia was experienced, nor the smallest degree of nausea; and even these disgorged matters were attempted to be masticated, although generally thrown out on account of the disagreeable taste.

In speculating upon the nature of this case, it appears evident that the energy of the digestive and assimilating organs were greatly diminished: consequently the stomach, deriving its influence, whether that presiding over the muscular action or vascular secretion, from the same source,—namely, the organic system of nerves,—experienced a proportionate diminution in its secreted juices. This was rendered apparent by the changes which took place in the aliments, when taken even in very moderate quantity, and when retained without being submitted to remastication.

Connected with debility of this organ, an increase of its animal sensibility, which it derives from the distribution of the eighth pair of nerves, appears to have been present. Under these circumstances, the gastric juices (being, as inferred, in diminished quantity,) could be sufficient only for a small portion of aliment, which nevertheless had been taken in an abundant quantity; and, having combined with that part whose state is most favourable to such an admixture, and being, by the usual action of this organ, conveyed to the pylorus, the imperfectly masticated portions, and that part which remains unpenetrated by the gastric juices, must either continue at the cardiac extremity, or be propelled there by the action of the stomach. That the undigested portions of the food do not only remain in that situation, but may, by a peculiar and complicated action of this organ, be conveyed there, may be proved not only by reasoning upon the nature of its organic action, but has even been demonstrated by large fistulæ of this organ, situated at its anterior convexity, and opening externally at the epigastric region. In a case in the Hôpital de la Charité, under the care of Corvisart, the complicated action of this organ was witnessed, conveying the digested portions of the ingesta towards the pylorus, which passed only in very small quantity, while the bulk of the unchanged aliment was propelled, by a contrary action, to the opposite extremity of the organ. It cannot be supposed improbable that the irritation produced in this part of the stomach by the unchanged aliments in ruminating individuals, should excite the animal sensibility of this organ; and if the brain be in a state capable of receiving the sensation, it is propagated to the organs of respiration, and their action induced through the medium of the same set of nerves,—namely, the par vagum,—that forms not only the respiratory class, but also the connecting chain between the organic and animal orders of the grand nervous system; and, while it bestows an exquisite sensibility on the pulmonary system, it likewise gives a requisite, but sparing, share of its influence to this important organ.

In effecting the process of rumination, the organic contractility of the stomach can do no more than, by an elective process (soon to be explained), place the aliments about to be returned in a situation, in respect to the cardia, favourable to the excitation of the animal sensibility of this organ, and to its ready regurgitation and propulsion along the œsophagus. So soon as the demand is made upon the sensibility by the situation of the alimentary bolus, the par vagal class of nerves is excited to action, and a full inspiration is effected, as has been described. The introduction of the bolus into the cardiac extremity of the œsophagus, may be considered as effected by the ordinary contractility of the stomach; perhaps sympathetically heightened at the moment by the re-action of the abdominal muscles; while, at the same time, the diaphragm has just undergone relaxation, in which the cardia may, from intimate nervous communication, suffer a similar participation, and thus give facility to the ascent of the alimentary ball in the œsophagus, which immediately contracts behind it from the irritation produced by its passage, and the bolus is thus conveyed to the mouth.

That relaxation of both the diaphragm and cardiac extremity of the œsophagus actually exists at the moment, although the glottis still remains closed, appears confirmed, both by the period of the respiratory act at which this process is produced, and from the circumstance that, when any restraint is exercised over this affection, it is principally by means of exciting the diaphragm to a frequent and continued action, when the premonitory sensation is felt at the cardia.

The influence of the will appears to be requisite, since the process is interrupted during sleep. But this influence is only passively engaged in the production of the ruminating act, by bringing about the co-operation of the respiratory organs.

The elective process exercised by the stomach in this affection, is similar to that which it employs in periods of health, and may be considered as relative to the degree of digestive energy, and to the comparative states in which the various ingesta may enter the stomach.

During the process of digestion, contraction takes place irregularly and in various situations in this organ, according as various portions of the longitudinal or circular fibres may act: this operates in producing a degree of arrangement in the aliments; and, as the gastric juices combine with the more soluble portion of the food, especially that situated towards the mucous surface of this organ, which, when duly effected, is conveyed, by the varying organic contractility of the muscular coat, towards the pylorus; while a successive and concentric stratum comes in contact with, and, if in a permeable state from its previous comminution and admixture with the salivary juices, is soon penetrated by, the secretions of this organ; and even the central mass not unfrequently is obliged to yield its more fluid parts to the exterior layer, when there is a deficiency of fluids in the alimentary contents. Hence the not unusual necessity for drink that takes place as digestion proceeds. In the course of this process, as it is the result of the healthy functions of the organ, the chyme in contact with its mucous surface is conveyed in a direction from the cardia to the pylorus. But, if the propagation of the digested contents towards this extremity of the organ proceed faster than it can pass through into the duodenum, the accumulation of chyme that consequently takes place in that direction tends to propel, and even to return, the less soluble portions towards the cardia; where, according to the state of the organ, it may produce cardialgia, acrid eructations, or even rumination.

In the debilitated state of the stomach, and consequent deficiency of the secretions, digestion can be perfectly performed only when the aliments are presented in small quantity, and in a favourable state, from complete comminution and from intermixture with the salivary juices. If, however, in this state of the organ, the food is conveyed rapidly into it, possessed of neither of these requisites, so as to produce sudden distension, a re-action of this viscus upon its contents takes place; and, as the imperfectly masticated food constitutes the greater portion of the ingesta, there is abundance present to be returned into the cardia, while there is a deficiency of aliment in a fit state to combine with, or to be operated upon, by the gastric juices; which, when effected, is rapidly conveyed to the other extremity of this organ, by the re-action of the muscular coat, from the undue distension and the stimulus of solid contents. Thus a double effect is produced by the healthy organic contractility of this organ, when in a weakened state, and yielding a diminished quantity of the usual fluids; which state, indeed, may be considered as constituting this peculiar affection,—namely, that part of the aliment which is dissolved by the gastric juices is conveyed towards the pylorus, while, at the same time, the tonic action tending to diminish the capacity of the organ pushes the less comminuted and indigestible portions of food into the unresisting cardia; which is returned, as I have attempted to describe, in order to undergo a second comminution and intermixture with the salivary juices; after which it is in a fit state to be conveyed to its destination along the mucous surfaces, with the juices of which it combines, and thus permits a central portion of the mass to return and undergo a similar process.

Having occupied the attention of the reader so long, perhaps with a dry disquisition, on the succession of phenomena that appear to me to constitute this peculiar affection, I shall now very shortly direct his attention to the general plan of treatment which I pursued; after which, I shall exhibit a hasty sketch of similar cases that have attracted my notice.

In the curative plan pursued during the time this gentleman was under my care, the cutaneous eruption was viewed as originating in the long and progressive derangements of the digestive canal; and the ruminating affection, from the highly intelligent history of its origin afforded me by the patient, as well as of the sensations and connexions of the phenomena so kindly accorded me during my attendance, was considered as the most advanced and peculiarly modified state of dyspepsia, or gastric debility.

With this impression, the indications that obviously presented themselves were correctly pursued. Having premised full evacuations of the intestinal and biliary organs, a course of tonics, combined with gentle and corroborative aperients, was entered upon, and pursued for some time, in conjunction with the warm bath and frictions of the whole surface twice or thrice a-week, and the usually-adopted stimulating ointments and washes to the diseased part of the cutis. Early in this tonic course, owing to the desire of my patient to have the cutaneous eruption as speedily removed as possible, I was induced to try the effect of the diaphoretics familiarly employed in similar affections. The various preparations of antimony were all found to increase the derangement of the stomach; guaiacum, either when combined with mezereon and sarsaparilla or with other medicines, produced the same effect; and the pilulæ submuriatis hydrargyri compositæ, which I have frequently employed with advantage in cutaneous diseases, produced in this case great gastric irritability.

Owing to the peculiar state of the digestive organs, the diaphoretic plan of treatment, which was entered upon merely to satisfy the desire of my patient,* was entirely abandoned; and no medicines whose direct operation upon the cutaneous expansions could be looked for, were employed, unless a pill of four grains of myrrh and one of ipecacuanha, given night and morning, as an auxiliary to a tonic draught in the middle of the day, may be viewed as operating in that manner; as no doubt it indirectly might.

Under the use of infusions made from a combination of vegetable tonics, aperients, and aromatics, with the addition of an alkaline carbonate or a carminative tincture, and the frequent use of the warm bath, with subsequent friction; while, at the same time, a deliberate mastication of the aliments, and a moderate indulgence in light and digestible food, was enjoined; amendment soon became apparent. After a fortnight's continuance of this plan of treatment, the cutaneous disease had made considerable progress towards removal, and the ruminating affection, which till then had been present after every considerable meal, was now very seldom experienced; nor did

* It is not always a pleasant duty for a physician to treat the diseases of one who has no mean opinion of his own medical knowledge, although he may not professedly be a medical man.

any symptoms of dyspepsia take its place, unless when the injunctions regarding the mode of living and mastication of the food were not attended to, or when subjected to causes operating a diminution of the digestive energy; then, dyspeptic symptoms, or even slight rumination, occasionally presented themselves.

Within a few weeks, the eruption was entirely removed; but the ruminating affection returned whenever the proper precautions were not observed. Having impressed the mind of my patient with the necessity of pursuing correctly the plan I had prescribed to him, upon the grounds that such an affection, if indulged, would, by impeding the nutritive functions, gradually undermine the energy of his system, he became more attentive to the state of his digestive organs and to his mode of living: and now, for several months, he has enjoyed perfect health, and had no return of the ruminating act. Having transferred, as he says, the gratification formerly enjoyed in the second mastication to the first, this process is now performed more deliberately; a more complete admixture of the aliments with the salivary fluids and with the air, takes place; while the stomach is less suddenly, and much more moderately, distended: the action of the bowels has also become more regular.

In exhibiting the history of our knowledge of this disease, it is a matter of difficulty to determine whether it was known to the ancients, and, if known, in what light they viewed the affection; for I can scarcely call it with justice a disease, seeing that its possessors (from the cases I am about to exhibit,) did not consider it such, from its being, on the contrary, rather attended with considerable enjoyment. If we consider the habits and boundless luxury of the civilized among the ancients, the manner in which the stomach was unloaded of a previous meal, in order to re-enter upon a second gratification of the palate, among the Grecian and Roman gourmands, in their respective eras of luxury, it may be easily inferred that such an affection as that which I have now exhibited, would have been considered a most delightful source of animal gratification; and certainly would not have been the less indulged, nor would the enjoyment have been diminished, had a similar opinion been entertained by their physicians as was propagated by honest Fabricius ab Aquapendente, that the possessor was endowed with a double stomach, and that the other bestial concomitants might, in process of time, be expected, either in themselves or their more bestial descendants.

Galen, who had ample opportunities of observation among the many instances of indigestion he must have met with in the luxurious, but peaceful, court of the Antonines, does not give the history of a single case,—(so far as the *Index* affords me the knowledge, for, in truth, it would take too much time to get through the text when indices can be obtained; and we all know that Galen wrote as much in his long life-time as would require an equal period of existence in many of our modern physicians to read;)—and, amid the various stomach-aches and affections of Marcus Aurelius, which, it would appear, both puzzled the brain and excited the anxiety of this prince of physicians and truly great man, so as to make him afraid that a glass of spiced wine might be too hazardous a remedy for the good emperor, the faculty of regurgitating his meals for a second mastication, appears not to have entered into the number. Unless we suppose that, not being considered a disease, the interference of Galen was not required, upon the ground that matters of taste, in the animal as well as in the mental application of the word, give a heightened enjoyment from their deliberate rumination.

Fabricius ab Aquapendente (*Op. Anat. Physiol.* p. 11, fol. 137,) furnishes two of the earliest instances of human rumination. The first is of a nobleman, in whom it generally took place an hour after his meals; which, whether solid or fluid, were always returned, in order to undergo a second mastication. Fabricius thought it proper to mention that the father of this individual had a horn growing from his forehead; and, with great good faith, adds, "ex quo forte datur nobis intelligi, parentis semen aliquam habuisse cum cornugeris animalibus, neque mirum fuisse genitum filium simile, quid a parente contraxisse:"—that, although the son did not inherit his father's horns, yet he possessed the accompanying faculty of rumination.

The second instance with which Fabricius has favoured us, was in a monk, who, although possessed of a most ravenous appetite, died of marasmus. This monk was possessed of still higher bestial attributes; for Fabricius describes him as having his forehead loaded with two horns; and Johannes Burgowerrus (*Dissert. de Rum. Hum.* Basil, 1626,) who visited this monk, in the company of Joh. Prevotius and Thos. Minadous, wrote a Dissertation on this interesting individual, and afforded Fabricius with the particulars which are inserted in his works. Burgower also adds, that the brother of this monk was also adorned with two budding horns, "Duorum cornuum vestigia gestasse," as a *striking* feature of family likeness; or, as this author will have it, "Quod enim fratris erat, id monacho ruminanti simul gratis impertiunt." But this illustrious individual did not ruminate, unhappily for the argument of Thos. Bartholinus, who, from these two individual instances, hastens to the conclusion (in his *Treatise de Unicornu*, cap. 2, and his

*Anat. Historiæ*, cent. 1, n. 39, et *V. Hist.* 16,) from the obvious analogy of the *cornugeræ pecudes*, that all human ruminants are possessed of horns; and also avers, that a double stomach will always be found on dissection. This piece of credulous doctrine cost the laborious Conrad Pyer, (in his *Merycologia*, p. 220,) no small trouble to refute; and he concludes in his turn, taking his honour to witness, (for he treats the subject with great gravity,) that this did not agree with his experience; seeing that many accounts have been given of horned individuals, none of whom ruminated.

Dan. Sennertus (*Med. Pract.* lib. iii. part i. sect. 11, cap. 8,) furnishes another history of a man of forty, who possessed this faculty from a child. He finds no difficulty in accounting for its occurrence, when he learnt that, when a child, this individual had lost his mother, and had been fed during his non-age with the milk warm from the cow. He accordingly soberly concludes, that he sucked it in with his nurse's milk: "Quamobrem deficiente educatione, cum orbus infans, et institutionis humanæ inops *nutricem vaccam* observaverit tuereturque attentius, ispse ruminationi paullatim assuevit, sodalitii familiaritate degenerans, &c."

Philip Salmuth, (in the first Century of his Observations, p. 100,) furnishes us with another instance of human rumination. It always took place in this individual about a quarter of an hour after having left table. He always ate ravenously, and swallowed his food almost without any previous mastication.

Joh. Faber Lynceus, (in *Expositione Histor. Nardi Anthonii Recchi*, p. 630,) gives an instance of most obstinate rumination in a highly respectable German, who, even when seated over his cups with his friends, was always obliged to retire about half an hour after the meal into a remote corner of the apartment, and then ruminated the ingesta, undisturbedly and as quickly as possible; which having done, he enjoyed uninterruptedly the society of his friends. "Having been asked how he became obliged to indulge this propensity, he answered that from a boy he had been subject 'to acid eructations;' that, after having reached his thirtieth year, he found it impossible to resist admitting into his mouth the food that constantly regurgitated from his stomach. And, being farther interrogated whether the second mastication of his food afforded him any gratification, 'Indeed,' he replied, 'it is sweeter than honey, and accompanied by a more delightful relish.'"

This affection might be said to be in the family of this honest German; for he was blessed with two grown sons: the older, of twenty-four years, also possessed this delightful faculty, but had it more under control than the father, as he could prevent it altogether when in company. The younger had not then come to its possession.

G. H. Velchius adduces another example, (in his *Observ. Med. Episag.* xxxvi.) in an inhabitant of London; who, in the fortieth year of his age, and of sound health, always returned his food, in order to undergo a slower and more deliberate mastication. Rumination always took place in this individual from an hour to two hours after a meal, and even at the remoter period it still preserved a pleasant taste, and was without any degree of acidity.

In an instance adduced by Daniel Ludovicus, (in the *Ephimerides Nat. Cur.* Decur. i. anno ix. and x. Observ. clx.) that occurred in a young woman, this act was not performed with the usual pleasure, and the returned food possessed a more disagreeable taste than that which accompanies perfect cases of this affection. Bitters and stomachic purgatives did not prevent its occurrence, which however was not always regular in its appearance; and, although cathartics and emetics prevented it for a short period, it soon returned. With all due respect for the memory of Dan. Ludovicus, I consider this to have been more allied to apepsia than to rumination.

Joh. Conrad Pyer, (*Merycologia*, lib. i. cap. vi. p. 69,) mentions a case in a fatuitous* young man; also its occurrence in a rustic in Switzerland; and in a woman in the neighbouring town. He sagely endeavours to prove, from the circumstance of those individuals being rustics and cowherds, that the frequent sight of the ruminating process had impressed their brains with a similar propensity, which, although imperceptible in its progress, had nevertheless ripened into maturity.

Slare, (in an early volume of the *Philosop. Trans.* the 193d article,) mentions the case of a Bristol man, who appears to have possessed this faculty in its perfection. This individual not only ruminated the solid ingesta, but also fluids, as milk and soups. There was, however, one imperfection connected with this case, as it relates to the state of this man's stomach during his meals; and which the very imperfect relation of the case (which was merely an answer to a number of queries transmitted from one of the Fellows of the Society in London, to an individual in the country, who merely contents himself with having answered the proposed questions,) furnishes this piece of information, that the victuals always seemed to descend imperfectly into the stomach, and to lie in the lower part of the throat. However, the portion of the meals first taken was first ruminated.

* I was lately informed, by a very intelligent individual, that a fatuitous boy resided in the village of Clapham a few years since, who constantly ruminated all his meals. This individual died at the age of fourteen.

In a case related in the 286th Number of the *Journal général de Medicine* by Mr. TARBES, as quoted by the Editor of the London Medical and Physical Journal, the prominent phenomena were nearly the same as the one I have just related.

Indeed, all the instances on record nearly agree in the individual phenomena constituting this affection, which, as it takes place by an unvarying mechanism, must, in its chief characters, remain unaltered, until it change into a different affection.

Dissections have not been able to throw any light upon this affection; nor can it be expected, in the present state of our medical knowledge, that, even in the event of a violent death taking place in a ruminating subject, that any visible alteration in structure could be detected. Fabricus ab Aquapendente and Thos. Bartholinus were confident of finding two stomachs, at least, in ruminating individuals, from the analogy of the cornuted tribes. Pyer, and Morgagni, (*de Sed. et Caus. Morb.* Epist. xxix. art. 4,) ridiculed the idea, and supported a contrary opinion, upon the ground that there were animals that ruminated without a double stomach.

The only instance upon record, to my knowledge, in which inspection after death took place, was in the instance of this affection occurring in a monk. This dissection is recorded both by Jo. Rhodius (Cent. ii. obs. 59,) and also by Bonetus, (in his *Sepulchretum*, l. iii. s. v. ob. 9 and 10:) it was made by Fanciscus Plazzonus, and is thus related by Jo. Rhodius:—"Monachus cum voluptate cibus ruminavit. Medici brutorum more gemino ventriculo prœditum putabant. Ipso defuncto, F. Plazzunos œsophagum reperit undiquaque carnosum instar musculi, reliquis universi corporis partibus se recte habentibus." The physicians of the seventeenth century were not much enlightened by the opening of this monk, but their dreams respecting the existence of two stomachs were henceforth dissipated.

2, *Walworth Terrace; March* 13, 1821.

